# Green monomeric photosensitizing fluorescent protein for photo-inducible protein inactivation and cell ablation

**DOI:** 10.1186/s12915-018-0514-7

**Published:** 2018-04-30

**Authors:** Yemima Dani Riani, Tomoki Matsuda, Kiwamu Takemoto, Takeharu Nagai

**Affiliations:** 1 Graduate School of Engineering, Osaka University, 1-3 Yamadaoka Suita, Osaka, 565-0871 Japan; 2 The Institute of Scientific and Industrial Research, Osaka University, 8-1 Mihogaoka, Osaka, Ibaraki 567-0047 Japan; 3 Graduate School of Medicine, Yokohama City University, 22-2 Seto, Kanazawa, Yokohama, 236-0027 Japan

**Keywords:** Photosensitizer, Superoxide, CALI, ROS, Cell ablation, Protein inactivation, Green fluorescent protein, Pleckstrin homology domain

## Abstract

**Background:**

Photosensitizing fluorescent proteins, which generate reactive oxygen species (ROS) upon light irradiation, are useful for spatiotemporal protein inactivation and cell ablation. They give us clues about protein function, intracellular signaling pathways and intercellular interactions. Since ROS generation of a photosensitizer is specifically controlled by certain excitation wavelengths, utilizing colour variants of photosensitizing protein would allow multi-spatiotemporal control of inactivation. To expand the colour palette of photosensitizing protein, here we developed SuperNova Green from its red predecessor, SuperNova.

**Results:**

SuperNova Green is able to produce ROS spatiotemporally upon blue light irradiation. Based on protein characterization, SuperNova Green produces insignificant amounts of singlet oxygen and predominantly produces superoxide and its derivatives. We utilized SuperNova Green to specifically inactivate the pleckstrin homology domain of phospholipase C-δ1 and to ablate cancer cells in vitro. As a proof of concept for multi-spatiotemporal control of inactivation, we demonstrate that SuperNova Green can be used with its red variant, SuperNova, to perform independent protein inactivation or cell ablation studies in a spatiotemporal manner by selective light irradiation.

**Conclusion:**

Development of SuperNova Green has expanded the photosensitizing protein toolbox to optogenetically control protein inactivation and cell ablation.

**Electronic supplementary material:**

The online version of this article (10.1186/s12915-018-0514-7) contains supplementary material, which is available to authorized users.

## Background

Elucidating protein function within cells is a major focus in molecular, cellular and developmental biology. Many methods are used to elucidate gene/protein function such as genetic knock-out and RNA interference (RNAi). However, genetic methods cannot be applied to essential genes during development or house-keeping genes in general since their loss will cause lethality. Also, to see the effect of RNAi requires lead time based on the turnover of the target protein [1].

Photosensitizers are chromophores which generate excessive reactive oxygen species (ROS) when irradiated by excitation light. An excited sensitizer may generate ROS via electron transfer to ground-state oxygen to generate superoxide anion radicals or energy transfer to ground-state oxygen to produce singlet oxygen [2]. Singlet oxygen (^1^O_2_) has a 3.5-μs lifetime in water and may diffuse in a 100-nm range [3], while the superoxide anion radical (O_2_^•-^), although not yet assessed, is known to be unstable and reacts immediately with iron-sulphur (Fe-S) clusters or undergoes a dismutation process [4]. As a result of the dismutation process, hydrogen peroxide (H_2_O_2_) has a relatively longer lifetime, ~ 1 ms with a diffusion range of several micrometers, and highly reacts with cysteine residue [4, 5]. A highly reactive and unspecific species, the hydroxyl radical (•OH), which is derived from H_2_O_2_, may diffuse over ~ 10 Å and has a 1-ns lifetime in water [6]. With these properties, when localized close to a target protein, the ROS may cause intramolecular or intermolecular cross-linking, aggregation or fragmentation of target protein [7]. Thus, photosensitizers can elucidate protein function in a specific spatial and temporal manner through chomophore-assisted light inactivation (CALI) which overcomes a major limitation of gene knock-down and RNAi [7, 8]. Photosensitizers are also often targeted to mitochondria, chromatin or plasma membranes to induce cell death [9, 10]. Thus, not only protein function, but also cell-specific function within a population could be elucidated by utilizing photosensitizers [11–13].

Currently, there are two types of photosensitizers: chemical dye-based ones (e.g. malachite green [8], fluorescein [14], eosin [15]) and genetically encoded ones. In order to inactivate specific target proteins, chemical photosensitizers need a targeting method such as antibody conjugation [7, 16] or a transgenically encoded tag with affinity for the modified photosensitizing ligand, e.g. FlAsH [17], ReAsh [18] or HaloTag [15]. These methods depend on the uptake of the exogenous CALI agent [19], the penetration of which may be problematic in thick specimens with multiple cell layers. However, fusing a genetically encoded photosensitizer directly to a target protein or subcellular localization signal expands the CALI technique, where spatiotemporal control is preserved without the need for chemical addition.

The first established genetically encoded photosensitizer, KillerRed, was derived from anm2cp protein [20]. KillerRed has been applied to elucidate protein function [21, 22], or cell ablation in vitro and in vivo [23, 24]. However, KillerRed tends to form homodimers at high concentration, and sometimes its dimeric property hampers subcellular localization [20] or affects cell division [25]. SuperNova, a monomeric variant of KillerRed, was developed, and proved to be superior to KillerRed as a fusion partner with signal peptides or localized proteins [25]. SuperNova itself has subsequently been useful for CALI purposes [26] and cell ablation [25].

Continuing from KillerRed and SuperNova, other photosensitizer colour variants were developed, KillerOrange and mKillerOrange (dimeric and monomeric orange variants of KillerRed respectively) [27] and a dimeric green variant of KillerRed [28]. In addition to the KillerRed-derived proteins, another monomeric green flavin mononucleotide (FMN)-based genetically encoded photosensitizer, miniSOG [29], and its improved variants singlet oxygen photosensitizing protein (SOPP) [30], SOPP2, SOPP3 [31] and miniSOG2 [13] have been described. However, the phototoxicity of miniSOG and its variants is dependent on the FMN concentration [9]. Unlike miniSOG, KillerRed-based variants do not depend on FMN concentration for phototoxicity since they have spontaneously forming green fluorescent protein (GFP)-like chromophores [32]. In certain applications, where the environment lacks FMN, KillerRed-based variants are preferable**.** Thus, in this study, we introduce the development of SuperNova Green, a green monomeric variant of a KillerRed-derived genetically encoded photosensitizer.

Since colour variants of genetically encoded photosensitizers exist, it has been suggested that combinations of photosensitizers which are activated at different excitation wavelengths should allow selective ablation of two different cell populations in a spatiotemporally controlled manner [12, 27]. The application is not only limited to whole cells; it can also be used to selectively inactivate different proteins within single cells using activating light which can be controlled precisely in space and time with subcellular resolution. Here, we demonstrate that ROS generation from SuperNova Green is specific to blue light irradiation and, therefore, compatible with concomitant use of the original SuperNova (hereafter called SuperNova Red), thus realizing advanced multi-colour CALI and cell ablation with up to micrometer and millisecond precision.

## Results

### Establishment and characterization of SuperNova Green

**Fig. 1:**
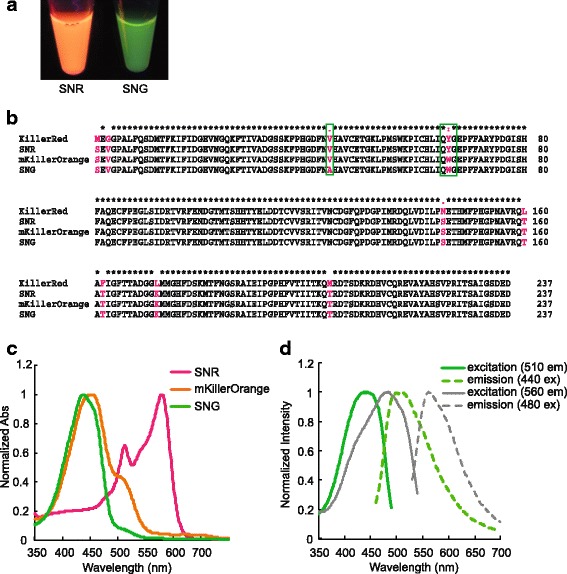
Establishment of SuperNova Green from SuperNova Red. **a** Protein solution of SuperNova Red (*SNR*) and SuperNova Green (*SNG*). **b** Sequence of SNG compared to its predecessors: KillerRed, SNR and mKillerOrange. SNG has tryptophan-based chromophore as seen in mKillerOrange and an additional Val44Ala mutation (*green box*). **c** Absorption spectra of SNR, mKillerOrange and SNG. SNG has absorbance peak at 437 nm and small shoulder at 510 nm. **d** Double excitation and emission spectrum of SNG. Excitation at 440 nm resulted in 510 nm emission (*green solid* and *dashed lines* respectively); excitation at 480 nm resulted in 560 nm emission (*grey solid* and *dashed lines* respectively)

To fill in the colour palette of KillerRed-derived monomeric variants, we developed SuperNova Green (SNG) from SuperNova Red (SNR). To do this, first we introduced the Y66W mutation into SNR to generate the orange variant of SNR, known as mKillerOrange [27], which has an absorbance peak at 453 nm with a shoulder at 510 nm. To further blue shift the absorbance peak, we introduced the V44A mutation reported previously to create a green variant of KillerRed [28]. As a result, V44A-mKillerOrange has an absorption peak at 437 nm with a smaller shoulder at 510 nm. We call this variant SuperNova Green (Fig. 1a–c). SNG has dual excitation/emission at 440/510 nm and 480/560 nm respectively with molar extinction coefficient 28,000 M^−1^ cm^−1^ and an absolute fluorescence quantum yield of 0.23 (Fig. 1d, Table 1).

**Table 1:** Protein characteristics of SNR and SNG

Protein	Abs. peak (nm)	λ_ex_ (nm)	λ_em_ (nm)	ε (M^−1^ cm^−1^)	Quantum yield
SNR	579	579	610	33,600	0.3
SNG	437	440	510	28,000	0.23
		480	560		

In V44A-KillerRed, the V44A mutation was suggested to prevent chromophore maturation to red, thus creating a green variant. De Rosny and Carpentier (2012) [28] described that the “E68 side chain in A44-KillerRed has two alternate conformations. One of which was in a catalytic position to promote green to red maturation similar to S69 in DsRed by catalyzing the C65 acylimine bond [33] while the other conformation was an inactive form” [28]. Based on that, we hypothesized that the double excitation and emission of SNG (Fig. 1d) possibly arises from the fractions with a completely matured orange chromophore and an intermediate green chromophore.

To test this, we compared the fluorescence intensity of SNG and mKillerOrange emission from a 0.05 μM protein solution under the same measurement conditions. Excitation of mKillerOrange at both 440 and 510 nm gave an orange emission (peak ~ 560 nm), while excitation of SNG at 440 nm resulted in green emission (peak ~ 500 nm) and excitation at 510 nm resulted in orange emission (peak ~ 560 nm) (Additional file 1: Figure S1a, b). This result supports the previous study [28] suggesting that the V44A mutation creates two forms of chromophore in SNG protein solution: the matured form of the orange chromophore and the intermediate green chromophore. However, when the fluorescence intensities of SNG excited at 440 and 510 nm were compared, the orange emission was ~ 40% of the green emission (Additional file 1: Figure S1b). Meanwhile, mKillerOrange showed greater fluorescence emission intensity when excited at 510 nm than when excited at 440 nm. We suspect therefore that the fraction of the matured orange chromophore in SNG is lower than the intermediate form which produces green emission. Also, mKillerOrange maturation to the orange chromophore apparently occurs more efficiently than in SNG. Because the dominant emission of SNG is generated with 440 nm excitation, this excitation light was used in subsequent experiments.

Following standard fluorescence protein characterization, the photostability of SNG to 440 nm light irradiation in 10 μM protein solution was investigated. The bleaching rate of SNG was compared to that of enhanced green fluorescent protein (EGFP) excited with 480 nm light at the same power density. The results showed that SNG bleaches faster than EGFP (Additional file 1: Figure S2). Indeed, correlation between photobleaching and ROS production has been assessed in other photosensitizers such as KillerRed and miniSOG. In KillerRed, rapid bleaching is associated with conversion of red chromophores to protonated green chromophores, and extensive photoirradiation will destruct the amino acid side chain at positions 65 and 66. Both are correlated to photosensitization of KillerRed [34]. For miniSOG, a positive correlation was found between photobleaching and increasing of ^1^O_2_ phosphorescence upon prolonged light irradiation [35]. Although correlation between photobleaching and ROS production is not extensively discussed in this manuscript, we found that investigation of this phenomenon would be useful to reveal the ROS-generation process, especially in the KillerRed-based variant with a tryptophan-based chromophore.

To confirm the oligomeric status of SNG, we used in vitro gel filtration chromatography with a 10 μM protein solution. As expected, this suggests that SNG maintains the same monomeric property as the parent protein, SNR (Additional file 1: Figure S3) and a standard monomeric fluorescent protein, mCherry. Although based on the marker position, it seemed that the SNR, SNG and mCherry size somehow shifted to > 29 kDa (when the expected size was ~ 27–29 kDa, we reproduced the data shown in [25] when SNR monomericity was confirmed using the ultracentrifugation method). Thus, we concluded that SNG maintained the SNR monomeric property. Meanwhile, as expected, KillerRed showed a peak near 75 kDa, which may mean that oligomerization occurs at a 10-μM protein concentration. To evaluate SNG as a fusion construct, it was fused to fibrillarin and vimentin in HeLa cells (Additional file 1: Figure S4a–h). The KillerRed and KillerRed-V44A fusions did not localize correctly, whereas the monomeric SNG and SNR both did. Furthermore, SNG localized correctly when fused to several proteins and subcellular localization signals, e.g. Lyn, histone 2B, actin and two tandem copies of mitochondrial localization signal (Additional file 1: Figure S4i–l).

### SNG enables cell ablation and dominantly produces ROS through electron transfer over energy transfer mechanism

**Fig. 2:**
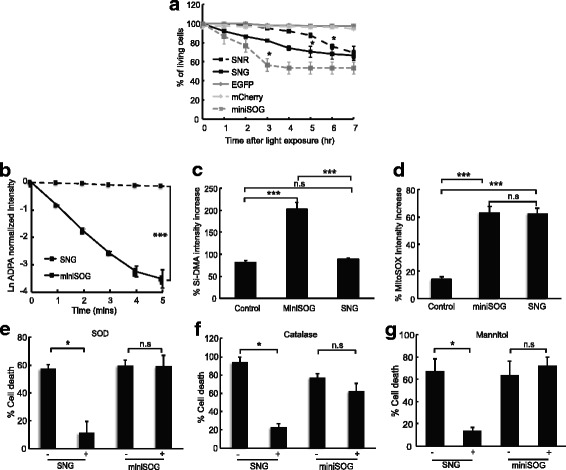
SNG photosensitization mechanism and phototoxicity in living cells **a** Phototoxicity of SNG (*black box*, *black solid line*) compared to SNR (*black box*, *black dashed line*) and miniSOG (*grey box*, *grey dashed line*) targeted to matrix mitochondria in HeLa cells. HeLa cell death was counted for 7 h every 1 h post-irradiation with 2 W/cm^2^ excitation light for 2 min. EGFP- (*grey diamond*, *grey solid line*) and mCherry- (*light grey diamond*, *light grey dashed line*) expressing cells are treated with the same power and time of irradiation as negative control for excitation light. Time for significant cell death to occur was analysed between each hour post-irradiation to t_0_. miniSOG, SNG and SNR showed significant cell death after 3 h, 5 h and 6 h respectively (*p* < 0.05, one-way analysis of variance (ANOVA), Tukey, *n* = 144 cells for SNR, 128 cells for SNG, 73 cells for miniSOG. Cells were calculated from 8 images per construct). No significant cell death occurred for EGFP and mCherry (*p* > 0.05, one-way ANOVA, Tukey, *n* = 117 cells for EGFP, 149 cells for mCherry. Cells were calculated from 8 images per construct). **b** Time course of SNG and miniSOG ^1^O_2_ measurement. miniSOG irradiation with 440 nm light caused significant ADPA bleaching compared to SNG (*p* < 0.001, *t* test, *n* = 4 replicates for miniSOG, 6 for SNG; each replicate came from independently purified protein samples). ^1^O_2_ (**c**) and O_2_^•-^ measurement (**d**) in HeLa cells expressing SNG or miniSOG in mitochondrial matrix using Si-DMA and MitoSOX respectively. After 10 s irradiation with 4 W/cm^2^ excitation light, significant Si-DMA fluorescence intensity increase was observed for HeLa cells expressing miniSOG (compared to control, independent *t* test, *p* < 0.001, *n* = 100 cells), but no significant difference observed between SNG and control (*p* = 0.185). MitoSOX fluorescence intensity increased significantly in cells expressing miniSOG and SNG (compared to control, independent *t* test, *p* < 0.001, *n* = 100 cells), but there was no significant difference between SNG and miniSOG (*p* = 0.659). **e**, **f** and **g** show O_2_^•-^, H_2_O_2_ and •OH quenching experiment on HeLa cells expressing miniSOG and SNG in mitochondrial matrix by 200 U/mL SOD, 1000 U/mL catalase and 60 mM mannitol respectively. HeLa cells expressing SNG treated (+) with SOD (**e**) and catalase (**f**) showed significant reduction in cell death after 2 W/cm^2^ excitation light irradiation for 2 min compared to non-treated (−) cells (*p* < 0.05, *t* test, *n* = 45 cells for SNG (−) SOD, 38 cells for SNG (+) SOD; 40 cells for SNG (−) catalase, 94 cells for SNG (+) catalase; cells were calculated from 4 images for each condition), while miniSOG showed no reduction in cell death (*p* > 0.05, *t* test, *n* = 59 cells for miniSOG (−) SOD, 62 cells for miniSOG (+) SOD; 67 cells for miniSOG (−) catalase, 93 for miniSOG (+) catalase; cells were calculated from 4 images for each condition). Same result was observed for mannitol experiment (**g**) (*p* < 0.05 for SNG, *t* test, *n* = 220 cells for SNG (−) mannitol, 185 cells for SNG (+) mannitol, 155 cells for miniSOG (−) mannitol, 180 cells for miniSOG (+) mannitol; cells were calculated from 4 images for each condition). Error bar represents ± standard error of the mean (SEM). Supporting numeric data are provided in Additional file 2

We next assessed the ability of SNG to induce cell death in HeLa cells. Figure 2 illustrates the SNG photosensitization mechanism and phototoxicity in living cells. Supporting numeric data for Fig. 2b, e–g are given in Additional file 2. SNG was targeted to the mitochondrial matrix of HeLa cells and then irradiated with 2 W/cm^2^ 440/60-25 nm excitation light for 2 min. Compared to HeLa cells expressing SNR, SNG caused cell death faster than SNR yet slower than miniSOG. No significant cell death was observed when 440/60-25 nm and 562/40 nm (2 W/cm^2^, 2 min) were applied to EGFP- and mCherry-expressing HeLa cells, indicating that irradiation by light itself does not cause cell death within this time frame (Fig. 2a).

To assess which ROS SNG produces, we examined ^1^O_2_ generation of SNG in comparison to miniSOG upon irradiation of 438/24 nm light. The protein solutions were mixed with a previously known ^1^O_2_ probe, anthracene-9,10-dipropionic acid (ADPA), and generation of ^1^O_2_ was measured by fluorescence decay of ADPA in a time-dependent manner [36]. Compared to miniSOG, which is known to produce both ^1^O_2_ and O_2_^•-^ [29, 35, 37, 38], the ^1^O_2_ generated from SNG was much lower (Fig. 2b). But when compared to KillerRed, SNR and also EGFP and mCherry as negative controls, SNG also did not produce a significant amount of ^1^O_2_ (Additional file 1: Figure S5a). ADPA alone did not bleach upon irradiation with 438/24, 475/28 and 575/25 nm excitation light (Additional file 1: Figure S5b). However, it was reported in [35] that ADPA also responds to O_2_^•-^, thus causing us to question the ADPA bleach of miniSOG in our experiment. It is also possible that ADPA sensitivity to O_2_^•-^ depends on the amount of O_2_^•-^ generated by the sensitizer. In that case, it may be that the O_2_^•-^ generated by KillerRed/SNG/SNR via our particular power density was not enough to significantly bleach ADPA as much as the ^1^O_2_ and/or O_2_^•-^ generated by miniSOG. To further confirm if SNG produces significant amounts of ^1^O_2_, we tested it using Si-DMA (silicone-containing rhodamine-9,10-dimethylanthracene), a far-red ^1^O_2_ sensor which only responds to ^1^O_2_, not to O_2_^•-^, H_2_O_2_ or other ROS [39]. The HeLa cells expressing SNG also did not produce significant amounts of ^1^O_2_ compared to normal HeLa cells, while cells expressing miniSOG did (Fig. 2c).

Since SNG does not work through an energy transfer mechanism, we speculated that SNG produces other ROS that are generated via electron transfer in keeping with its parental proteins SNR and KillerRed [25, 34, 40]. To test this hypothesis, we used MitoSOX, a hydroethidine (HE)-based redox probe that was conjugated to a triphenylphosphonium group (TPP^+^) to target it to mitochondria. HE itself would react specifically with O_2_^•-^ to form 2-hydroxyethidium (2-OH-E^+^) or other ROS (H_2_O_2_ and nitric oxide), peroxidase or redox-active metal ions (iron or copper) to form the non-specific product ethidium (E^+^) which would emit red fluorescence. However, it was shown previously that HE does not react with ^1^O_2_ [38]. ROS generation in HeLa expressing miniSOG or SNG in the mitochondrial matrix shows a significant red fluorescent increase of MitoSOX compared to the control, and yet no significant difference was found between miniSOG and SNG (Fig. 2d). Here, we conclude that SNG and miniSOG produce the same amount of ROS other than ^1^O_2_ when applied in mammalian cells.

Since it is widely known that O_2_^•-^ is the primary ROS generated through a photosensitization process, we performed a quenching experiment on O_2_^•-^ and its product from the dismutation process, H_2_O_2_, and •OH, which is generated from H_2_O_2_ through Fenton’s reaction. HeLa cells expressing SNG or miniSOG in the mitochondrial matrix were treated with 200 U/mL superoxide dismutase (SOD), then the number of cell deaths was compared between irradiated and non-irradiated cells. A significant decrease in cell deaths was observed from cells expressing SNG but not miniSOG (Fig. 2e). The same results were observed for cells treated with 1000 U/mL catalase to scavenge H_2_O_2_ (Fig. 2f) and 60 mM mannitol to quench •OH (Fig. 2g). These substances could limit the ability of SNG to cause cell death, while they had no effect on miniSOG-expressing cells. We assume that ROS generated by miniSOG cannot be scavenged by SOD, catalase or mannitol because the type of ROS generated by miniSOG is dominated by singlet oxygen.

Based on this study, we speculated about how SNG could be used to mediate CALI and cell ablation. O_2_^•-^ as the primary ROS produced from excited chromophores might attack a protein target immediately due to its proximity to that protein. In a situation where mitochondria are loaded with the photosensitizers, we may assume that O_2_^•-^ is produced excessively, and it may undergo dismutation to H_2_O_2_ and then Fenton’s reaction to •OH. Our treatment with the scavengers discussed previously may help reduce the effect of ROS which may lead to cell death. However, since scavenging O_2_^•-^ and H_2_O_2_ does not produce a totally non-reactive species, the mechanisms by which SOD and catalase could save the cells from cell death remain open questions. A more quantitative and specific measurement of each ROS produced by SNG using a more specific sensor would help to reveal those mechanisms. Nevertheless, from our results we conclude that SNG produces unaccountable amounts of ^1^O_2_ and more likely to produce O_2_^•-^ and its derivatives through electron transfer mechanism.

### Selective light irradiation results in selective pleckstrin homology domain inactivation

**Fig. 3:**
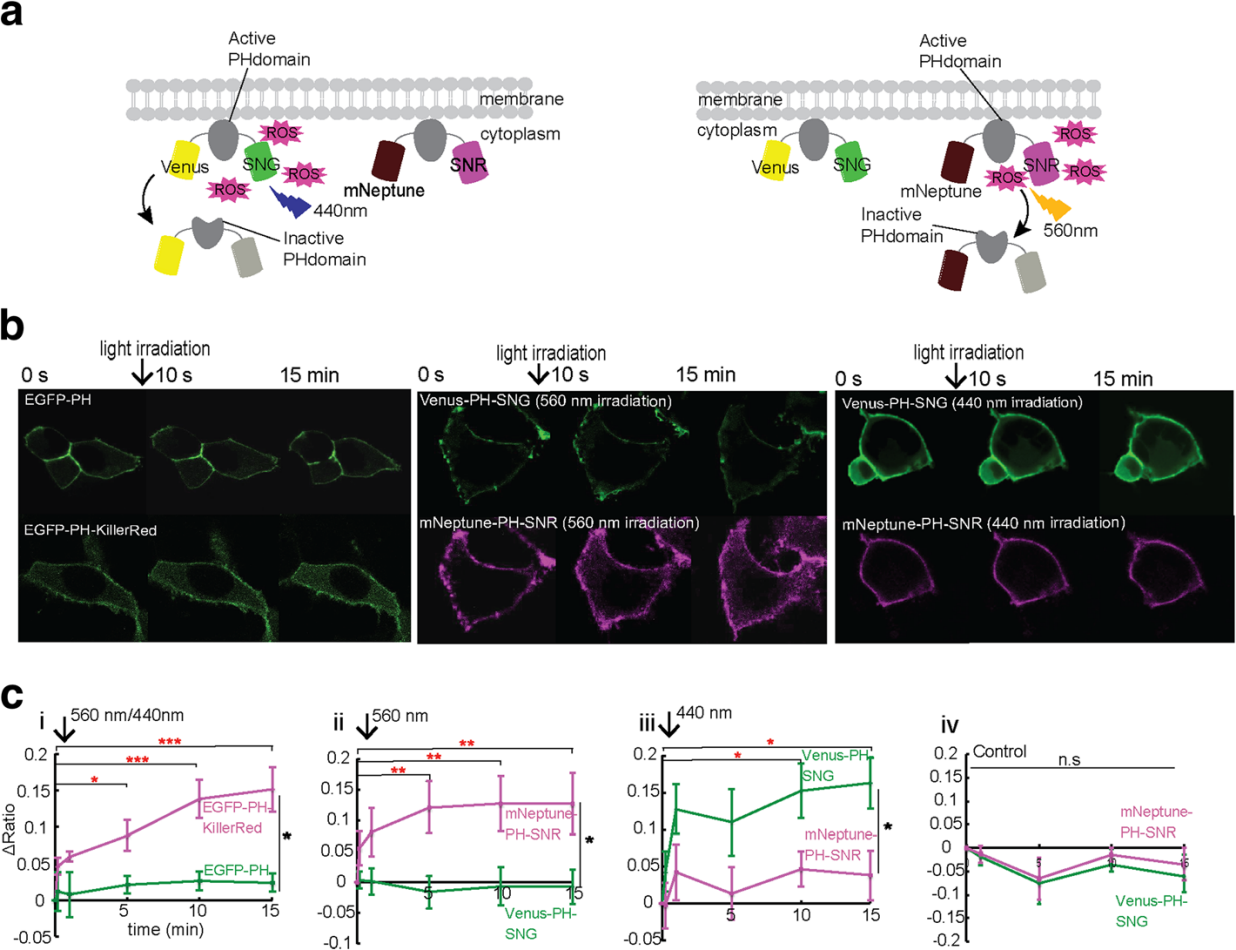
Demonstration of selective CALI. **a** Schematic overview of a selective CALI experiment using two indicator constructs with distinct excitation to induce ROS production which then liberates them from a plasma membrane tether. **b** Images taken at 0 s (before ROS-producing light irradiation), 10 s (immediately after light irradiation) and 15 min after 3 W/cm^2^ light irradiation for 10 s. Prior to ROS-producing light irradiation, all constructs were localized to the plasma membrane. After inactivation of the PHdomain, fluorescence increase in cytoplasm was seen for EGFP-PH-KillerRed (560 nm irradiation), mNeptune-PH-SNR (560 nm irradiation) and Venus-PH-SNG (440 nm irradiation). **c** Quantitative measurement of cytoplasm-to-plasma membrane fluorescence ratio increase. Fluorescence ratio of cytoplasm and plasma membrane at each time point was normalized to *t*_0_. As negative and positive control respectively, EGFP-PH (440 nm) and EGFP-PH-KillerRed (560 nm) were used (i). Only EGFP-PH-KillerRed showed a significant ratio increase (*p* < 0.05, one-way ANOVA, Tukey, *n* = 10 cells). (ii) 560 nm light irradiation to co-transfected cells with Venus-PH-SNG and mNeptune-PH-SNR caused a significant ratio increase of mNeptune fluorescence over time (*p* < 0.05, one-way ANOVA, Tukey, *n* = 11 cells) and also when compared to Venus at *t*_15_ (*p* < 0.05, *t* test, *n* = 11 cells for each construct). Conversely, 440 nm light irradiation (iii) caused significant ratio increase of Venus fluorescence (*p* < 0.05, one-way ANOVA, Tukey, *n* = 10 cells) and when compared to mNeptune at *t*_15_ (*p* < 0.05, *t* test, *n* = 10 cells for each construct). (iv) Images of light control taken from cells expressing Venus-PH-SNG and mNeptune-PH-SNR without light irradiation were calculated and showed no significant ratio changes over time. Error bar represents ±SEM

We next tested if ROS generation by SNG could inactivate a target protein specifically upon blue light irradiation and tested the concept of its compatibility (or not) when concomitantly used with a red photosensitizer (SNR) which generates ROS upon orange light irradiation for multi-colour CALI. We used SNG and SNR to inactivate the pleckstrin homology domain (PHdomain) of phospholipase C-delta 1 (PLC-δ_1_), which binds inositol 1,4,5-tris-phosphate, abbreviated Ins(1,4,5)P_3_, in the plasma membrane [41]. The expected result was that selective photosensitizer-mediated ROS generation by blue or orange light would cause PHdomain detachment from PLC-δ_1_, liberating a fluorescent probe into the cytosol. We constructed mNeptune2.5-PHdomain-SNR and Venus-PHdomain-SNG. mNeptune2.5 and Venus allow distinct visualization before and after PHdomain inactivation caused by the responsible photosensitizer, SNR or SNG respectively (Fig. 3a). EGFP-PHdomain and EGFP-PHdomain-KillerRed were used as negative and positive controls respectively.

Images of HEK293 cells expressing EGFP-PHdomain taken at time 0 show localization of EGFP to the plasma membrane. The cells were then irradiated with 3 W/cm^2^ 440 nm light for 10 s. After light irradiation, images were taken at 10 s, 1 min, 5 min, 10 min and 15 min. The cytoplasm/membrane intensity ratio for cells expressing the EGFP-PHdomain construct did not change after light irradiation, suggesting that EGFP did not cause PHdomain inactivation and that 440 nm light irradiation itself did not affect the target protein. On the contrary, as previously reported by Bulina and colleagues [41], EGFP-PHdomain-KillerRed-expressing cells showed an increase in cytoplasm/membrane intensity ratio after 560 nm light irradiation (Fig. 3a, b, c, (i)).

We then irradiated cells co-expressing mNeptune-PHdomain-SNR and Venus-PHdomain-SNG with 3 W/cm^2^ 560 nm light for 10 s to inactivate the PHdomain by SNR. Images were taken in the Venus and mNeptune channels. After light irradiation, we could observe a significant increase in the mNeptune ratio but not the Venus ratio (Fig. 3c, (ii)). When we irradiated co-transfected cells with 3 W/cm^2^ 440 nm light to inactivate the PHdomain by SNG, the opposite was observed. There was no significant increase of the mNeptune ratio but a significant increase of the Venus ratio. The slight increase of the mNeptune ratio observed after 440 nm light irradiation (Fig. 3c, (iii)) might be due to the small absorbance peak of SNR at 440 nm (Fig. 1c). A light control experiment of cells expressing both Venus-PHdomain-SNG and mNeptune-PHdomain-SNR without light irradiation showed that imaging using a 488 nm and a 633 nm laser did not significantly affect the sensitizers (Fig. 3c, (iv)). This selective application of CALI suggests that the technique can be performed without significant collateral damage to the non-target protein, even when they are both in the same place—in this case the plasma membrane.

### Selective light irradiation results in selective cancer cell death

**Fig. 4:**
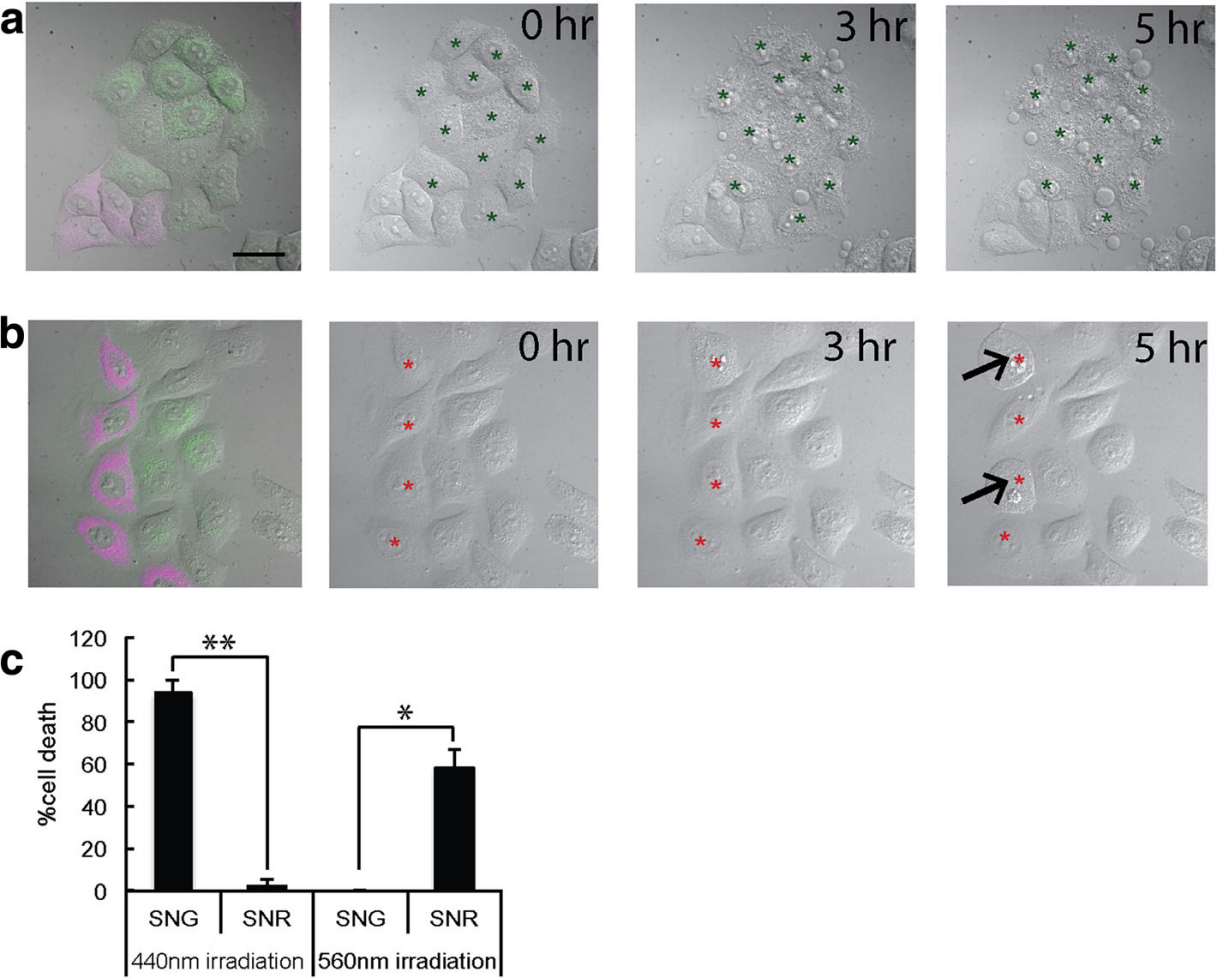
Selective cell ablation of co-cultures of HeLa cells stably expressing SNR and SNG in mitochondria **a** Co-cultures were irradiated with ~ 4 W/cm^2^ blue light for 2 min. Images were taken 0, 3 and 5 h post-irradiation. Only cells expressing SNG underwent cell death; cells expressing SNR survived. **b** Co-cultures were irradiated with ~ 4 W/cm^2^ orange light and images were taken as in **a**. Half of cells expressing SNR underwent cell death during this interval, while all cells expressing SNG survived. **c** Quantitative analysis for selective cell ablation with 440 nm and 560 nm light irradiation. Under 440 nm light irradiation, significant cell death occurred for cells expressing SNG compared to SNR (*p* < 0.01, *t* test, *n* = 97 cells). For 560 nm light irradiation, significant cell death occurred for cells expressing SNR compared to SNG (*p* < 0.05, *t* test, *n* = 36 cells). Scale bar = 20 μm. Error bar represents ±SEM


Next, we examined whether SNG could induce selective cell death while preserving the viability of SNR-expressing cells. For this, we generated stable HeLa cell lines expressing SNG or SNR in the mitochondrial matrix. Then, we co-cultured those two stable cell lines. When irradiated with 447/60-25 nm light at 4 W/cm^2^ for 2 min, 94% of cells expressing SNG were successfully ablated while almost 100% of the SNR-expressing cells survived (Fig. 4a, c, Additional file 3: Movie S1). On the other hand, when the co-culture was irradiated with 562/40 nm at 4 W/cm^2^ for 2 min, only cells expressing SNR were ablated; the cells expressing SNG survived (Fig. 4b, c). In this experiment, we noticed that SNG’s killing efficiency was higher than SNR’s, in keeping with the previous observation (Fig. 2e). Increasing the orange light power density did not increase the killing efficiency of SNR, but it did cause unspecific cell killing due to light toxicity (data not shown). We suggest that the SNR killing efficiency could be improved to achieve the same efficiency as that of SNG. In summary, this result indicates that SNG can be used in combination with SNR to ablate different populations of cells.

Additional file 3:**Movie S1.** Selective cell ablation for SNG irradiated by 440 nm light. (AVI 19889 kb)


## Discussion

The preceding results show that a green variant of SNR, SNG, has been developed and characterized. SNG is the first green monomeric photosensitizing protein derived from KillerRed. Currently, there are three colour variants of the KillerRed-based photosensitizer: red, orange and green. By using these three colour variants, inactivation can be achieved by irradiating samples with 560 nm, 510 nm and 440 nm light respectively. We show here that irradiation by 510 nm light did not ablate cells expressing SNG but did kill cells expressing mKillerOrange (Additional file 1: Figure S5). This means that three-colour variants of genetically encoded photosensitizers may be possible to use in combination.

As previously mentioned, although miniSOG, an FMN-derived genetically encoded photosensitizer also generates ROS when irradiated at 440 nm, in some conditions where the FMN cofactor is absent or sparsely present, the miniSOG phototoxicity is ineffective [9]. Here we show that SNG produces predominantly O_2_^•-^ (and its derivatives). Unlike SNG, the original miniSOG produces both O_2_^•-^ and ^1^O_2_ [35, 37, 38], whereas improved iterations of miniSOG (SOPP [30], SOPP2 and SOPP3 [31]) are directed towards complete ^1^O_2_ generation.

O_2_^•-^ and its derivatives H_2_O_2_ and •OH, which are known to be produced naturally in normal cells through NADPH oxidase, complex I and complex III reductase in mitochondria, have several specific targets to maintain physiological functions. O_2_^•-^ is highly reactive towards Fe-S clusters and is known to modulate transcription factors in bacteria [5]. H_2_O_2_, due to the properties we have mentioned in the Background section, is speculated to play a role in many cellular events, e.g. maintaining cellular redox potential, Ca^2+^ homeostasis and apoptosis [42]. Since moderate changes in the concentrations of O_2_^•-^ and its derivatives may control intracellular events [43], applications of SNG beyond protein inactivation or cellular ablation may extend into the exploration of subcellular ROS function in intracellular events.

We realized that several endogenous chromophores are effective photosensitizers, such as riboflavin and FMN with absorption peaks ~ 450 nm and ^1^O_2_ quantum yields (Φ_Δ_) of 0.49 [44] and 0.65 (in D_2_O) respectively [37]. Therefore, we thought that irradiation with blue light might reduce the spatiotemporal resolution and ROS selectivity of SNG photosensitization compared to green/orange light irradiation. However, since studies using miniSOG have shown achievable spatiotemporal inactivation due to focused and proximate ROS generation to a protein target or cellular compartment [1, 45, 46], we suppose that inactivation with a certain power density would not be achieved from a free endogenous chromophore.

In some cases when fluorescence-based imaging (e.g. for Ca^2+^, voltage and cell cycle indication) is needed in combination with a photosensitizer to allow cause and effect to be visualized simultaneously, a shorter excitation wavelength photosensitizer would be more useful. As most fluorescence-based imaging requires a longer period of imaging compared to a photosensitizing process, photosensitizing is better achieved at shorter excitation wavelengths with visualization of the effect at longer excitation wavelengths [47]. For that purpose, we expect that SNG might be a more suitable tool than other KillerRed-based variants.

Some optogenetic tools have been used to manipulate cell behaviour and inactivate protein function by an anchor-away method (anchoring the target protein far away from its target/functional region in the cell) [48] or with photo-induced oligomerization by cryptochrome 2 (CRY2)/cryptochrome-interacting basic-helix-loop-helix (CIB1) proteins [49]. Compared to those systems, which fuse both CRY2/CIB1 components to the protein target and inhibitor/translocalization signal independently, inactivation by SNG is more straightforward and only requires fusion to a specific protein. Additionally, when longer term loss of function is favoured, irreversible inactivation by SNG would be preferred to the reversible CRY2/CIB1 inactivation in a setting where the dissociation rates are faster than the protein turnover rate.

## Conclusion

In conclusion, we showed here that SNR in conjunction with SNG enables selective cell ablation and CALI, expanding the toolbox for optogenetics. Together with other colour variants, SNG will be useful to elucidate multiple protein functions as well as cellular functions at the single cell or organism level with a high spatiotemporal specificity.

## Methods

### Vector construction

SuperNova Green was generated from SuperNova/pRSET_B_. To introduce point mutations at Y66 and V44A, direct mutagenesis was performed using the inverse polymerase chain reaction (PCR) method. V44A-KillerRed and mKillerOrange were established by introducing V44A and Y66W to the KillerRed-pRSET_B_ and the SuperNova/pRSET_B_ plasmid respectively using the same method. miniSOG was cloned from a miniSOG-C1 (Addgene #54821) plasmid into pRSET_B_ using the *Bam*HI-*Eco*RI restriction site.

For mammalian cell expression, PCR-amplified miniSOG, SNG, mCherry and EGFP were cloned into 2xCOXVIII-SNR/pcDNA3.1 by *Bam*HI and *Eco*RI restriction enzyme digestion. Fusion with vimentin was performed by replacing Vimentin-Kohinoor/pcDNA3.1 (Addgene #67772) with SNG, SNR, KillerRed and KillerRed V44A using *Bam*HI and *Eco*RI restriction enzyme digestion. The fibrillarin construct was made by replacing SNR in SNRΔ11-Fibrillarin/pcDNA3.1 with KillerRed, KillerRed V44A and SNG using the *Hin*dIII/*Bam*HI restriction site. Lyn-SNG/pcDNA3, SNG-H2B/pcDNA3.1 and LifeAct-SNG/pcDNA3 were made by replacing OeNL in Lyn-OeNL/pcDNA3.1 (Addgene #89528), Nano-lantern-H2B/pcDNA3 (Addgene #51971) and Kohinoor-Actin/pcDNA3.1 (Addgene #67776) with SNG at the *Bam*HI-*Eco*RI restriction site.

For selective CALI constructs, the PHdomain sequence was obtained from GFP-C1-PLCdelta-PH (Addgene #21179). Fusion proteins were generated using the restriction sites *Age*I-Venus-*Bgl*II-PHdomain-*Xba*I-SNG-*Eco*RI and *Age*I-mNeptune-*Bgl*II-PHdomain-*Xba*I-SNR-*Eco*RI and then cloned to C1 plasmids. All oligonucleotides used in this experiment are listed in Additional file 1: Table S1.

Plasmids were transformed into XL-10 Gold *Escherichia coli* cells (Agilent Technologies, Santa Clara, CA, USA) using the heat shock method. A single colony was picked and cultured in 1.5 LB medium containing 0.1 mg/mL carbenicillin and then processed for plasmid purification. The DNA sequences of mutants were confirmed by dye terminator sequencing using a Big Dye Terminator v1.1 Sequencing Kit (Applied Biosystems, Foster City, CA, USA).

### Protein purification

pRSET_B_ containing a gene encoding protein tagged with N-terminal polyhistidine tags was transformed into JM109 (DE3) (Promega, Madison, WI, USA) by heat shock transformation at 42 ^o^C for 45 s. The transformants were then plated onto agar plates containing 0.1 mg/mL carbenicillin. Colonies were cultured in 200 mL LB media containing 0.1 mg/mL carbenicillin at 23 °C with gentle shaking at 80 rpm for 4 days. Polyhistidine-tagged proteins were purified by Ni-NTA agarose (Qiagen, Hilden, Germany) chromatography, then eluted using 200 mM imidazole in TN buffer (10 mM Tris-HCl pH 8, 150 mM NaCl). The eluted proteins were processed with buffer exchange chromatography using a PD-10 column (GE Healthcare, Chicago, IL, USA). The final elution was diluted in 50 mM 4-(2-hydroxyethyl)-1-piperazineethanesulphonic acid (HEPES)-KOH (pH 7.4).

### Spectroscopy

Protein concentrations were measured using an alkaline denaturation method. Protein purity was confirmed using sodium dodecyl sulphate-polyacrylamide gel (SDS-PAGE) analysis. Absorption spectra were measured on a V630-Bio spectrophotometer (JASCO, Easton, MD, USA). The absorbance peak was used for the molar extinction measurement. The molar extinction coefficient was defined by the equation ε = *A*/*c*, where ε is the molar extinction coefficient at the absorbance peak, *A* is the absorption at the peak wavelength and *c* is the protein concentration.

For the fluorescence spectrum measurement, the protein was diluted until absorption at the peak wavelength was 0.05. The fluorescence spectrum was measured using an F7000 fluorescence spectrophotometer (Hitachi, Tokyo, Japan). The emission spectrum was measured using 380, 400, 420, 440, 480 and 510 nm as excitation wavelengths. Meanwhile 490, 510, 540, 560, 580 and 610 nm were used for the emission wavelengths.

To measure the quantum yield, the protein was diluted to 5 μM. The absolute quantum yield of the protein was measured using a Hamamatsu Photonics C9920-01 spectrometer (Hamamatsu Photonics) at 610 and 510 nm for SNR and SNG respectively.

#### Size exclusion chromatography

Size exclusion chromatography was performed with a Superdex75 100/300GL column (GE Healthcare) with ÄKTA explorer 10S (GE Healthcare). We injected 1 mL of 10 μM protein into the column and then eluted it with 10 mM HEPES and 100 mM NaCl, pH 7.2. Elution was performed at 1 mL/min.

#### Photobleaching assay

An SNG and EGFP 10 μM protein solution was placed in a silicone microwell (1–2 mm in diameter) and topped with a cover glass. Protein solutions were exposed to 17 W/cm^2^ of 447/60-25 nm (Brightline) and 475/42-25 nm (Brightline) excitation light for SNG and EGFP respectively using a mercury arc lamp as the light source. Images were taken every 10 min for 8 h. The fluorescence intensity from the images was measured using Metamorph software (Molecular Devices, San Jose, CA, USA). Curve fitting and determination of *t*_1/2_ were done using Origin Software (OriginLab, Northampton, MA, USA).

#### Cell culture, transfection and localization imaging

HeLa cells (RIKEN BRC, Ibaraki, Japan) and HEK293T cells (ATCC, Manassas, VA, USA) were cultured with Dulbecco’s modified Eagle’s medium (DMEM)/F12 with phenol red (ThermoFisher Scientific, Waltham, MA, USA) supplemented with 10% fetal bovine serum (FBS) (Biowest, Riverside, MO, USA). Cells were incubated at 37 °C with 5% CO_2_. For subculture, cells were washed with sterile phosphate-buffered saline (PBS) and dissociated with trypsin. Cells subjected to plasmid transfection were seeded on 3-mm glass bottom dishes, and the DNA was transfected using either the calcium phosphate method or Lipofectamine 2000 (Invitrogen, Carlsbad, CA, USA). For all live imaging experiments performed here, after 48 h of transfection, the medium was changed to DMEM/F12 without phenol red (ThermoFisher Scientific) added with 100 μg/mL penicillin/streptomycin (ThermoFisher Scientific). Imaging of subcellular localization was done using a confocal microscope (FV1000, Olympus) with a 60× NA 1.4 oil immersion objective. Images were taken using a 450 nm and a 580 nm multi-argon ion laser.

### Singlet oxygen and superoxide measurement

In vitro ^1^O_2_ generation was measured using ADPA (anthracene-9,10-dipropionic acid) (Molecular Probes). We added 10 μL of 1 mM ADPA to 50 μL of 50 μM protein diluted in 50 mM HEPES-KOH, pH 7.4. Then 15 μL of the mixture was diluted in 300 μL PBS (the final concentrations of ADPA and protein were 7.9 μM and 2 μM respectively) and placed in a cuvette. The solutions were irradiated with 47 mW/cm^2^ excitation light (438/24, 475/28 and 542/27 nm) from light engine spectra (Lumencor, Beaverton, OR, USA). The fluorescence intensity of ADPA (ex/em = 350/430 nm) was measured every 1 min using an F7000 fluorescence spectrophotometer (Hitachi).

To assess ^1^O_2_ generation of SNG and miniSOG, pcDNA3.1 plasmids encoding sensitizer protein with mitochondria translocalization signal were transfected to HeLa cells using the calcium phosphate method. Then 48 h after transfection, the cells were incubated with 25 nM Si-DMA (Dojindo, Kumamoto, Japan) in DMEM/F12 without phenol red (ThermoFisher Scientific) for 45 min at 37 °C. The cells then were irradiated with 4 W/cm^2^ 447/60-25 nm (Brightline) excitation light from a mercury arc lamp for 10 s. Images before and after irradiation were taken at the Cy5 channel (633 nm laser) with a Nikon A1 confocal system (Nikon, Tokyo, Japan). For O_2_^•-^ generation, HeLa cells expressing SNG or miniSOG were incubated with 1–2 μM MitoSOX (ThermoFisher Scientific) in DMEM/F12 without phenol red for 10 min and then irradiated with excitation light, as in the Si-DMA experiment, for 30 s. Images before and after irradiation were taken at the mCherry channel (with a 543 nm excitation laser). The fluorescence increase was measured in the same region of interest within the cells using NIS Elements Software (Nikon).

Quenching experiments were done on HeLa cells expressing SNG and miniSOG plated on 96-well plates. Cells were treated with 200 U/mL polyethylene glycol (PEG)-SOD (Sigma Aldrich, St. Louis, MO, USA), 1000 U/mL catalase from bovine liver (Wako) or 60 mM mannitol (Wako, Tokyo, Japan) in DMEM/F12 without phenol red for 1 h and then irradiated under a fluorescence microscope (Nikon Eclipse TE2000, oil immersion 40× Plan Apo objective lens, NA 1.4) with 2 W/cm^2^ 447/60-25 nm (Brightline) excitation light from a light engine (Lumencor) for 2 min. Differential interference contrast (DIC) images were taken using an ORCA-Flash 4.0 Camera (Hamamatsu Photonics, Hamamatsu, Japan). At least two images were taken from two independent dishes, and then cell death was enumerated 6 h post-irradiation.

#### Phototoxicity in mammalian cells

To assess phototoxicity in mammalian cells, pcDNA3.1 plasmids encoding SNR, SNG, miniSOG, mCherry and EGFP were transfected to HeLa cells (plated on a 35-mm glass bottom dish) using the calcium phosphate transfection method. At 48 h post-transfection, cells were irradiated on a fluorescence microscope (Nikon Eclipse TE2000-E, oil immersion 60× Plan Apo objective lens, NA 1.4) with 2 W/cm^2^ excitation light from an Intensilight C-HGFIE (Nikon). The filters used were 447/60-25 (Brightline), 475/42-25 (Brightline) and 562/40 (Brightline) for SNG/miniSOG, EGFP and SNR/mCherry respectively. DIC images were taken using an ORCA-Flash 4.0 (Hamamatsu Photonics). At least four different images were taken from each dish, and at least two dishes per construct were analysed for this experiment. The percentage of cell death was calculated every 1 h for 6 h post-irradiation.

### Selective cell ablation

To perform selective cell ablation, stable cell lines expressing SNR and SNG independently inside a mitochondrial matrix were generated by transfection of SNR/SNG-pcDNA3 to HeLa cells using Lipofectamine 2000. Selection was then performed using 400 μg/mL Geneticin (Invitrogen). Cells expressing SNR and SNG were then co-cultured in 30-mm glass bottom dishes and subjected to light irradiation of 447/60-25 nm and 562/40 nm at ~ 4 W/cm^2^ (mercury arc lamp). Cells were irradiated for 2 min, then DIC images were taken at 0, 3 and 5 h post-irradiation using an ORCA-Flash 4.0 (Hamamatsu Photonics).

#### Selective CALI

HEK293 cells were co-transfected with Venus-PHdomain-SNG/C1 and mNeptune-PHdomain-SNR/C1 using Lipofectamine 2000. At 48 h post-transfection, imaging was performed with a confocal microscope (A1 Nikon Confocal, Nikon Eclipse Ti). Light irradiation was performed using an Intensilight (Nikon) with ~ 3 W/cm^2^ 447/60-25 and 562/40 nm excitation under a 60× oil immersion objective lens NA 1.4 (Nikon) for 10 s. Images of Venus and mNeptune fluorescence were taken using a 488 nm laser and a 633 nm laser. Image analysis was performed using the QuimP plugin [50] in Fiji Software [51] to measure the fluorescence intensity in the cytoplasm and plasma membrane of cells. The ratio increase presented in Fig. 3c was calculated as:

$$ \Delta Ratio=\frac{Icytoplasm{t}_x}{Imembrane{t}_x}-\frac{Icytoplasm{t}_0}{Imembrane{t}_0} $$

where *t*_x_ refers to the time point after light irradiation and *t*_0_ is the time point before light irradiation.

#### Statistical analysis

Data fitting and statistical analysis were performed using Origin 8 software (OriginLab) and SPSS statistics (IBM). Statistical values including the exact *N* and statistical significance are reported in the figure captions.

## Additional files

Additional file 1: **Figure S1.** Emission spectra of SNG and mKillerOrange resulting from 440 nm and 510 nm excitation. **Figure S2.** Photobleaching curve of SNG and EGFP. **Figure S3.** Gel chromatography results. **Figure S4.** SNG monomeric property in mammalian cells. **Figure S5.** Control experiment of ^1^O_2_ measurement by ADPA. **Figure S6.** Selectivity between SNG and mKillerOrange upon 510 nm light irradiation. **Table S1.** List of oligonucleotides used in this article. (PDF 962 kb)

Additional file 2: Numeric data for Fig. 2b, e–g, Additional file 1: Figures S5, S6 (XLS 71 kb)

## Abbreviations

2-OH-E^+^: Dihydroxyethidium (2-hydroxyethidium)
ADPA: Anthracene-9,10-dipropionic acid
CALI: Chromophore-assisted light inactivation
CIB1: Cryptochrome-interacting basic-helix-loop-helix
CRY2: Cryptochrome 2
E^+^: Ethidium
FMN: Flavin mononucleotide
HE: Hydroethidine
PHdomain: Pleckstrin homology domain
PLC-δ_1_: Phospholipase C-delta 1
ROS: Reactive oxygen species
SNG: SuperNova Green
SNR: SuperNova Red
SOD: Superoxide dismutase
SOPP: Singlet oxygen photosensitizing protein
TPP^+^: Triphenylphosphonium
