# Supplementary material for: Green monomeric photosensitizing fluorescent protein for photo-inducible protein inactivation and cell ablation
Source: BMC Biol. 2018 Apr 30;16:50. doi: 10.1186/s12915-018-0514-7 (PMC5928576; doi:10.1186/s12915-018-0514-7)
Supplement: Supplementary file 2 — Figure S1. Emission spectra of SNG and mKillerOrange resulting from 440 nm and 510 nm excitation. Figure S2. Photobleaching curve of SNG and EGFP. Figure S3. Gel chromatography results. Figure S4. SNG monomeric property in mammalian cells. Figure S5. Control experiment of 1O2 measurement by ADPA. Figure S6. Selectivity between SNG and mKillerOrange upon 510 nm light irradiation. Table S1. List of oligonucleotides used in this article. (PDF 962 kb) [file 12915_2018_514_MOESM1_ESM.pdf]

Supplementary data

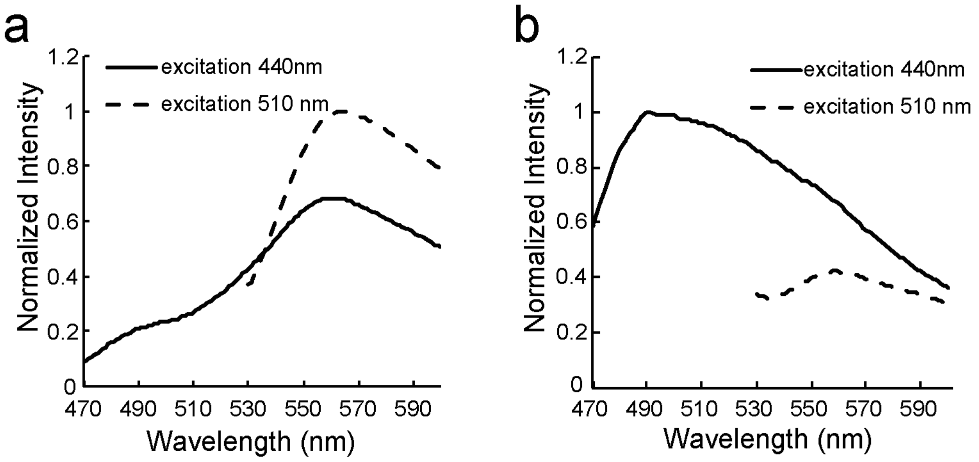

Figure S1. Emission spectrum of SNG and mKillerOrange resulted from 440 nm and 510 nm excitation. (a) Excitation of mKillerOrange at 440 nm and 510 nm both result in a 560 nm emission peak. (b) Excitation of SNG at 440 nm resulted in ~500 nm emission peak while excitation at 510 nm gave a~560 nm excitation peak. The green emission intensity of SNG was 2.5 fold higher than the orange emission.

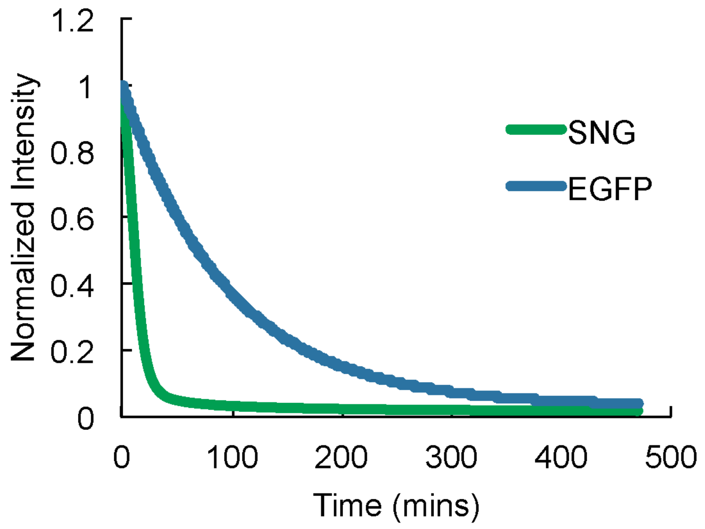

Figure S2. Photobleaching curve of SNG and EGFP. SNG was irradiated with 440 nm excitation light ($\tau_{1/2}$ = 10 mins) and EGFP ($\tau_{1/2}$ = 70 mins) with 480 nm excitation light.

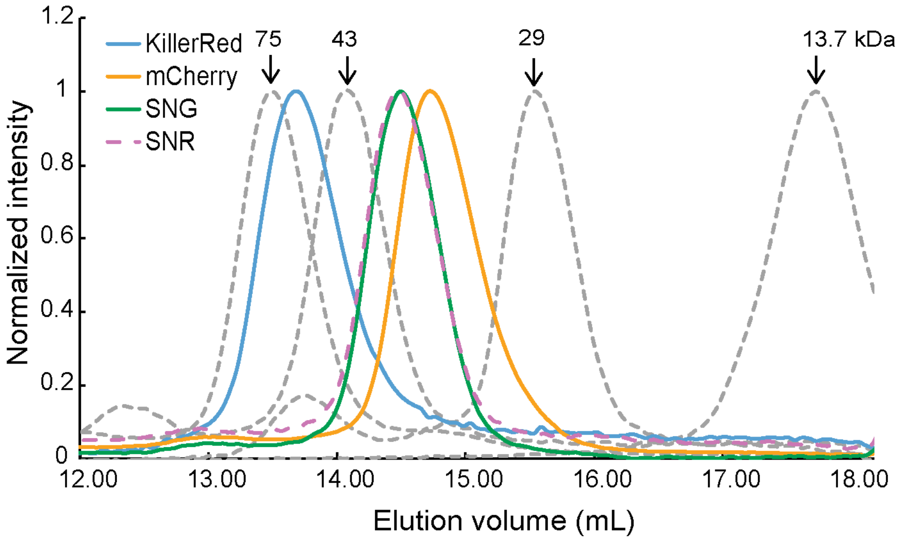

Figure S3. Gel chromatography result. KillerRed (~29 kDa) formed a dimer at 10 uM protein concentration meanwhile SNG and SNR elute as monomers together with mCherry as monomer control. 75 kDa (Canalbumin), 43 kDa (Ovalbumin), 29 kDa (Carbonic anhydrase) and 13.5 kDa (Ribonuclease A) were used as marker (dashed grey line).

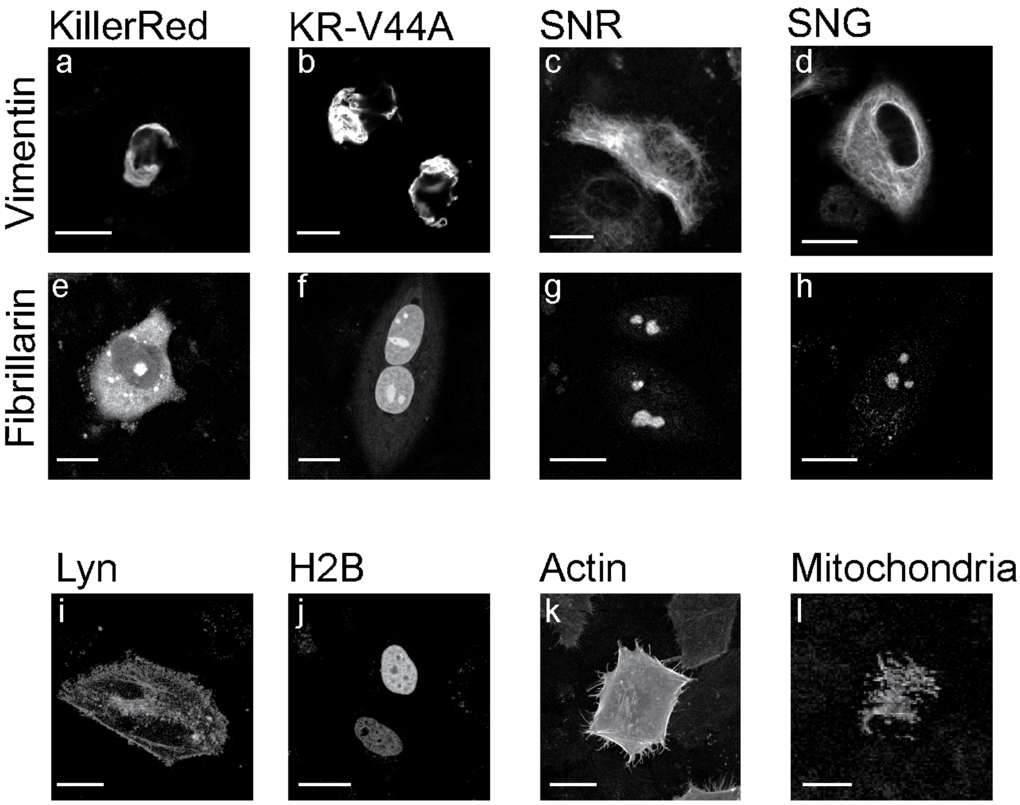

Figure S4. SNG monomeric property in mammalian cell. Comparison of KillerRed, KillerRed V44A (KR-V44A), SNR and SNG fused with Vimentin and Fibrillarin of HeLa cells (scale bar: 20 μm) (a-h). SNR and SNG showed correct localization to all target proteins tested meanwhile KillerRed and KR-V44A did not. (i-l) fusion of SNG to lyn, histone 2B, actin and tandem copies of mitochondria localization signal. Scale bar=20 μm.

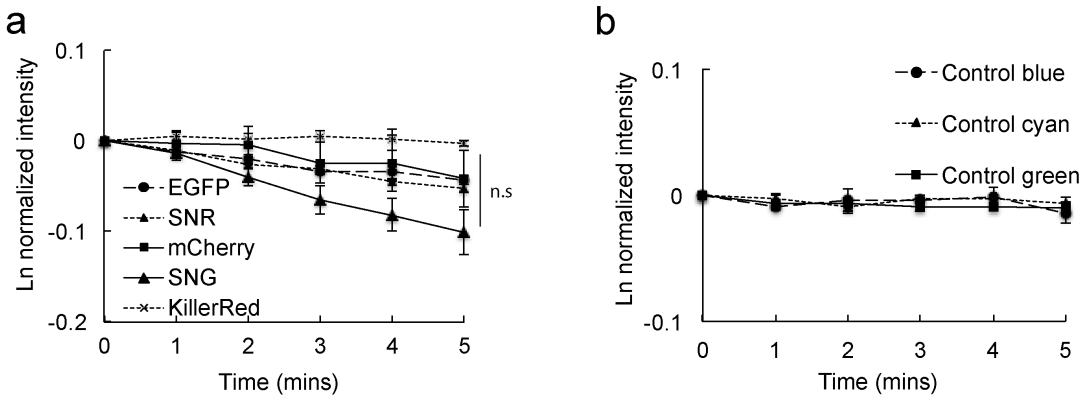

Figure S5. Control experiment of $^{1}O_2$ measurement by ADPA (a) Compared to KillerRed, SNR, EGFP and mCherry as a negative control, SNG did not cause significant ADPA bleaching ($p>0.05$, one-way ANOVA, n = 3 replicates for KillerRed, 4 for SNR, 4 for EGFP and 4 for mCherry, each replicate came from independently purified samples). (b) 7.9 μM ADPA diluted in PBS buffer was irradiated with 438/24 and 575/25 nm as light control (n = 3 replicates for each control). No significant ADPA fluorescence decrease occurred. Supporting numeric data is provided in Additional file 2.

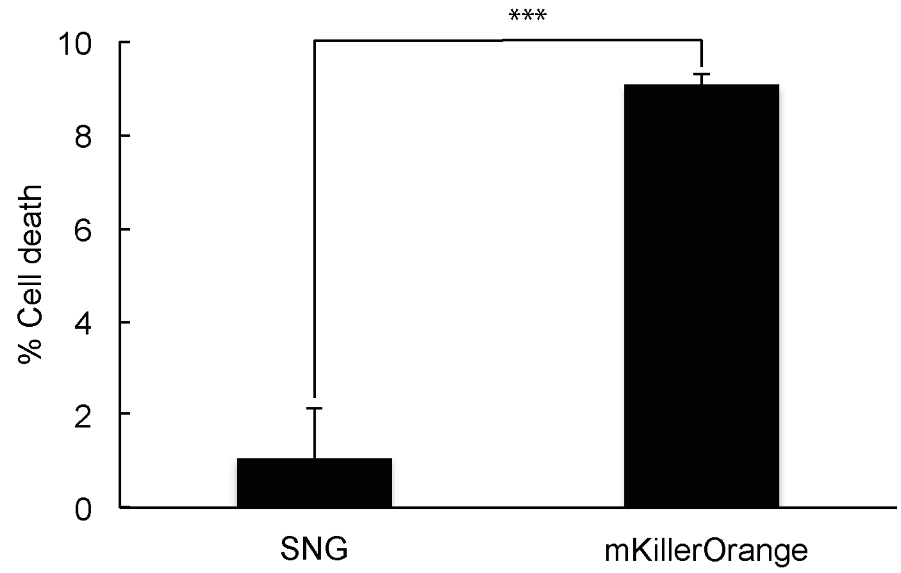

Figure S6. Selectivity between SNG and mKillerOrange upon 510 nm light irradiaiton. SNG and mKillerOrange phototoxicity in HeLa cells after light irradiation with ~1W/cm^2 510 nm for 2 mins (t-test, $p<0.01$, n = 141 cells for SNG, 89 cells for mKillerOrange. Cells were counted from 4 images for SNG and 3 images for mKillerOrange). Supporting numeric data is provided in Additional file 2.

Table S1. List of oligonucleotides used in this article

Y66W Forward primer	5'-TGG GGC GAG CCC TTC TTC-3'
Y66W Reverse primer	5'-CTG GAT CAG GTG GCA GAT GGG-3'
V44A Forward primer	5'-GCG CAC GCC GTG TGC GAG-3'
V44A Reverse primer	5'-GCG CAC GCC GTG TGC GAG-3'
BamHI-SuperNova Forward primer	5' - T TAG GAT CCG ATG GGT TCA GAG GTC GGC-3'
BamHI-SuperNova Forward primer (2)	5'-GC GGA TCC ATG GGT TCA GAG GTC GGC CCC-3'
EcoRI-stop codon-SuperNova Reverse primer	5'-GC GAA TTC TTA ATC CTC GTC GCT ACC GAT-3'
BamHI-miniSOG Forward primer	5'-A ATG GAT CCG ATG GGA AAA GAG CTT TG-3'
EcoRI-stop codon-miniSOG Reverse primer	5'-AAT GAA TTC TTA TCC ATC CAG CTG CAC-3'
HindIII-SuperNova Forward primer	5'-TA AAG CTT ATGG GTT CAG AGG TCG GC-3'
BamHI-stop codon-SuperNovaΔ11 Reverse primer	5'-TA GGA TCC GGG CAC GCT GTG G-3'
EcoRI-SuperNova Reverse primer	5'-GTA GAA TTC TTG ATC CTC GTC GCT ACC GAT GGC-3'
AgeI-kozak-Venus Forward primer	5'-T ATA CCG GTC CGC ACC ATG GTG AGC AAG GGC GAG-3'
BglII-linker-Venus Reverse primer	5'-TA AGA TCT GAG TCC GGA CTT GTA CAG CTC GTC CAT GCC GAG-3'
XbaI-SuperNova Forward primer	5'-AAG TCT AGA ATG GGT TCA GAG GTC GGC C-3'
AgeI-kozak sequence-mNeptune Forward primer	5'-A TAC CGG TCC ACC ATG GTG TCT AAG GGC GAA-3'
BglII-mNeptune Reverse primer	5'-GC TCT AGA TTA ATC CTC GTC GCT ACC G-3'
